# Widening Access: what do dental school websites signal to prospective students?

**DOI:** 10.1038/s41415-023-5454-0

**Published:** 2023-01-27

**Authors:** Michal M. Kawecki, Philip M. Newton

**Affiliations:** Swansea University Medical School, Swansea University, Swansea, SA2 8PP, UK

## Abstract

**Introduction** Widening Access (WA) policies aim to ensure that a professional workforce reflects the community that it serves by facilitating the admission of applicants from under-represented demographics. WA has not been extensively studied in UK dental education. Website discourses are an important element in students' higher education choices and have the potential to engage those who might be under-represented.

**Methods** Critical discourse analysis was used to investigate contents of the 16 UK dental school webpages in relation to WA, based on a previous study within medical education. Data were contextualised through identification of drivers and levers, as well as warrants of WA.

**Results** Discourses emphasising social mobility, and the resultant advancement within social hierarchy of an individual, dominated the narrative rationalising WA as an initiative. WA was depicted as a mechanism to support applicants of high academic ability and exhibiting commitment to studying dentistry but who were unable to show their true potential due to their underprivileged backgrounds. This presentation portrayed dental schools as generous establishments, selectively granting career-advancement opportunities to disadvantaged students. Discourses on the benefits of WA for patient care and workforce diversification were largely absent.

**Conclusions** Discourses representing WA on websites of UK dental schools are limited and do not portray applicants from deprived backgrounds or under-represented groups as individuals bringing unique positive benefits to dentistry and patient care. We encourage dental schools to consider alternate messages for attracting applicants from under-represented demographics.

## Key points

- Widening Access is a key initiative to ensure that the dental workforce represents the patients that it serves.
- Widening Access does so by encouraging recruitment of groups who are under-represented.
- Dental schools appear to be committed to Widening Access but they present a restricted view of how it can be achieved, emphasising academic criteria but overlooking the benefits that a diverse workforce brings to the profession.

## Introduction

Widening Access (WA) refers to an initiative which concentrates on providing opportunities for individuals from disadvantaged and under-represented groups to enter higher education (HE) and achieving their full potential.^1^^,^^2^

WA is unique to each country, depending on its historical and contemporary social challenges. For instance, in the USA, WA is oriented towards admission of learners from minority ethnic (ME) groups, while Canadian and Australasian medical schools are occupied with recruitment of students from indigenous populations or rural regions.^3^^,^^4^ In the UK, WA-initiatives are focused on learners from disadvantaged socioeconomic backgrounds (SEBs) (for example, deprived regions), different ethnic origins, or various demographic characteristics (age, gender).^1^ Barriers for certain social groups not transitioning into HE include: unequal availability of opportunities to attain high results in secondary school examinations; inability to pay private tuition fees; geographic locations; specific social and cultural capitals; entrenched familial and sociocultural expectations; or limited access to relevant information, guidance and advice.^5^

Contextual admissions (CA) help higher education institutions (HEIs) to identify applicants whose true abilities are not necessarily reflected in their formal qualifications and for whom HEIs can adjust their formal entry requirements.^6^ CA policies often necessitate the use of supplementary information pertinent to an individual's background features (for example, socioeconomic indicators, general school ranking) which are analysed together with their academic attainment levels. There is no universal algorithm through which establishments implement WA or use CA processes.^5^

The admissions processes employed by medical and dental schools shape the future composition of the workforce.^7^ Historically, both types of schools admitted a significant proportion of students from higher SEB,^8^ with dentistry being portrayed as a 'white' profession for the 'rich'.^9^ It is clearly competitive; in 2013, 1,190 students were admitted from 11,490 applications, representing a 10.4% acceptance rate.^10^ However, a recent review of medical and dental learners' ethnicity by Higher Education Statistics Agency (HESA)^11^ showed that 60.3%, 25.9%, 4.7%, 4.5% and 1.6% of students (n = 55,975) came from white, Black, Asian or ME groups, or 'unknown' backgrounds, respectively, roughly mirroring the ethnic composition of the country. Interestingly, this trend is not reflected among clinical academics. The Dental Schools Council's 2018 *Survey of dental clinical academic staffing levels* report revealed that 75%, 25% and 3.9% of staff came from white, ME or unknown backgrounds, respectively.^12^

Dental applications from learners of low SEBs remain low, in spite of efforts from HEIs to widen access and participation.^9^ Under-represented applicants can be demotivated from applying to a dental school by academic, financial, mentor-related, or institutional factors. High entry requirements for admission constitute an important challenge.

Analysis of attainment distribution pattern at A-level standard shows that 13% of privately-schooled pupils achieved grades placing them in the top decile nationwide (A, A, B). In contrast, only 2% of state-schooled pupils who had been receiving free school meals (a marker used in CA) achieved such attainment levels.^13^

Fourteen UK dental schools employ the University Clinical Aptitude Test (UCAT), which measures cognitive attributes, allowing schools to identify the most suitable candidates during their admission processes.^14^ However, UCAT can favour some candidates^15^ and the degree to which it could compensate for lower qualifications is equivocal.^16^ Importantly, evidence underpinning its use in dentistry is still emerging.^16^

A Bachelor of Dental Surgery degree (BDS) lasts two years longer than a standard Bachelor-level qualification and this can pose significant financial pressures, particularly for learners from deprived SEBs.^17^ Historically, dental students had an opportunity to receive free education alongside government grants meeting their living costs. Currently, learners can only apply for NHS bursary in their fifth year of study, while the first four years are funded through the student loans system, thus leading to accumulation of a substantial debt.^18^

WA attempts to create a diverse workforce, reflecting the patients they serve.^19^ Importantly, patients from lower SEBs tend to utilise health services to a higher extent than those from affluent SEBs.^20^ Deprived regions often struggle with professional recruitment and become underserved. However, healthcare professionals from disadvantaged SEBs are more likely to work in parallel regions serving similar patients after qualifying^21^ and undertake under-supplied careers.^22^ Availability of more diverse cohorts of clinicians can encourage access among patients who might otherwise feel culturally isolated.^23^ The aforementioned studies suggest that an increased recruitment of students from lower SEBs potentially brings many additional positives to the profession.

Work of Razack *et al.*^24^ and Alexander *et al.,*^25^ exploring discourses present on medical school websites, demonstrated that nature of such digital discourses might have a substantial effect on attracting diverse learners, as more than 90% of applicants often base their application decisions on the information contained on a particular school website.

We are unaware of any previous study looking at the discourses concerning WA on dental school websites in the UK and there is a general paucity of scholarship investigating WA within dentistry, unlike in medicine.^9^ It thus seems prudent to explore nature of the message that is communicated to prospective students about reasons for which they ought to consider dentistry.^26^

## Methods

This study is based on the conceptual framework and methodology derived from research of Alexander *et al.*^25^ and Razack *et al.*^24^^,^^27^^,^^28^ who employed critical discourse analysis (CDA) and the Foucauldian approach to analyse WA discourses on medical school websites. The Foucauldian approach aims to explore discourses in operation (within contextual framework) by revealing and bringing them out of the unconscious shadow domain.^29^ The Foucauldian perspective also looks at effects discourses produce on thinking, feelings and actions of individuals.

### Corpus

We replicated the approach undertaken by Alexander *et al.*^25^ but using the websites of UK dental schools. Overall, 16 universities offering a BDS degree were identified using the General Dental Council's website of registerable qualifications in dentistry. All UK qualifications leading to registration as a dentist were considered, apart from two programmes tailored specifically for qualified doctors or overseas dentists. All the websites and the content they referred to were incorporated into the current study. The final assembled corpus contained 52,041 words. Data were collected between June and August 2021. All the hyperlinks pertinent to entry criteria, admissions or WA were followed. The details of the dental programmes, and sample quotes, are located in the supplementary materials.

### Analysis

The data analysis included:

- Familiarisation with the specific website content of each UK dental school
- Contextualisation within a broader background of a school website
- Text collection from UK dental school websites. This incorporated information on: WA; Widening Participation; outreach activities and dedicated alternative access routes (for example, 'Foundation to Health and Veterinary Studies [Dentistry]' [Year 0]); or tailored degree programmes (for example, enhanced support degrees). The analysis was centred on the linguistic aspect at macro level
- Identification of all references (both implicit and explicit) to WA. Each statement was systematically investigated through the prism of style, structure, tone and evaluative language employed. This helped to explore the construction and presentation of WA by individual institutions and to compare differences and similarities between the respective portrayals.

CDA employed Hyatt's (2013)^25^^,^^30^ analytical framework. The following attributes were analysed:

- Drivers, which constituted goals of a policy
- Warrants, constituting justifications for a policy
- Levers - instruments of a policy enactment.

The above characteristics were located (as themes) and identified via a line-by-line analysis of the written material.

Textual analysis was performed using NVivo (v.11; QRS International). Consistent with Alexander *et al.,*^25^ one author (MK) undertook the primary analysis, with findings developed and refined through critical discussion with a second author (PN). Formal ethical approval was not required as the study did not involve any participants and all the materials were within the public domain.

## Results

In total, 11 of 16 dental schools contained information pertinent to WA directly on their websites. Four schools did not include WA material directly, but forwarded readers to relevant information through hyperlinks. One school which did not mention their WA approach was not included in the analysis.

A summary of the analysis is presented below together with representative quotes.

### Drivers

All schools communicated, either directly or by a strong inference, that the purpose of their WA policy was to provide students from disadvantaged backgrounds with an opportunity to study dentistry. However, this message only disclosed *'*what' WA aimed to do (augmenting numbers of students coming from disadvantaged or under-represented backgrounds, enhancing diversity of learners), rather than revealing its future benefit to the profession and patient care (hence its *'*value'). The terminology used *in lieu* of WA by various HEIs differed and included: gateway; contextual admission; widening participation; access programmes; outreach activities; and enhanced support degrees:

- 'The Enhanced Support Dentistry Programme is an opportunity for talented students from non-selective state schools in [redacted] to pursue a career in dentistry. Students receive additional academic support while following the standard five-year dentistry BDS course'.

### Warrants

The justification for WA policy was rarely mentioned. When included, it tended to refer to the importance of an individual's context, rarely addressing causative factors which could impact on performance of certain applicants. This also did not convey the positive impact a diverse group of dental students may have on the dental profession itself and quality of patient-management:

- 'Achieving a strong set of school or college grades is key to earning a place at our university. That can be challenging, and we know grades are influenced by things like where you live, and other factors. That's why each year we look at each student application in detail. We note circumstances that may have impacted your potential. We treat everyone fairly and admit the brightest and best students, regardless of background'.

### Levers

Only three schools mentioned governmental policy enactment as a reason for WA implementation. This also failed to overtly communicate the benefits of WA:

- 'The School of Dentistry is committed to increasing widening participation for its programmes in line with the dental school council's *Selecting for excellence* report'.

Overall, the ultimate aim of the WA policy and enhanced opportunities for prospective students were echoed well and clearly. The value and benefits of this initiative to wider communities was underemphasised, though.

### WA, academic meritocracy and social mobility

Policies tended to place individual applicants at the heart of the WA initiative. For instance, drivers for WA were frequently equated with identification of 'able' applicants and their subsequent provision with 'special' opportunities to be selected during an admission process to read a BDS degree:

- "The University of [redacted] is committed to Widening Access and ensuring that all students with the potential to succeed, regardless of their background, are able to apply with us. The additional information gained through contextual data enables our Admissions Advisory Panel to recognise student's achievements and identify their potential to succeed in the context of their background and experience'.

### WA, dental workforce diversification and development

Few statements challenged the prevalent discourse of WA for meritocracy and resultant mobility within social hierarchy. Two briefly highlighted the positives of a diverse student community and expressed the value of WA in terms of a general educational and social benefit to a wider community as a warrant:

- 'The University of [redacted] believes a diverse student population is important from an educational and social perspective'.

### Relationship of prospective WA applicants and dental schools

Within the prevailing discourse (WA for social promotion), WA was portrayed as granting opportunities to applicants who would historically be unlikely to consider reading for a BDS degree and as creating unique prospects with compensatory engagement opportunities which allow non-traditional learners to become more competitive in the admission process:

- 'The aim of widening participation is to make sure the faculty's student body is as diverse as possible by supporting students from backgrounds where people do not generally go onto a university education'.

## Discussion

This work identified a dominant stance: emphasising the value of WA to an applicant's receipt of opportunities for social advancement and social justice. This reflects the increasing individualism in UK HE, where students become consumers responsible for choices pertinent to their education and HE serves as a career-progression instrument.^31^

Discourses reflect what is considered desirable and valuable within a community. Here, the prevalent discourse reinforced anticipation that individuals should exhibit characteristics traditionally favoured among applicants. These were high academic attainment and appropriate extra-curricular activities. WA schemes allowed HEIs to apply dispensations to those applicants to compensate for their challenging SEBs, albeit their degree varied and was generally minimal (one- or two-grade reductions to enter dentistry). Consistently, WA was depicted as a student selection tool, aiding HEIs to recruit the 'worthy' and 'best-fitting' learners from a broader range of applicants. In this instance, WA functions as a corrective factor during merit-based evaluation and subsequent comparison between individual candidates.

The current findings resonate with those of Alexander *et al.* and Cleland *et al.,*^25^^,^^32^ who also demonstrated that academic meritocracy is a well-established belief held within HEIs in the UK. They found that discourse on WA for social advancement (promotion of learners with lower attainment levels) is often interwoven with another theme - academic, merit-based recognition within student recruitment systems (recognition of those with the highest attainment levels). These two unreconciled as-yet and competing approaches were also identified here.

Work in Canada by Razack *et al.*^24^^,^^27^^,^^28^ proposed that prominence and dominance of discourses on academic excellence and meritocracy can lead to a lesser degree of inclusion with healthcare professions. Similarly, a specific emphasis on academic credentials on admission webpages or policy documents accessible to the public could discourage applicants to schools in the UK. The UK admissions agency Universities and Colleges Admissions Service only allows four choices of medical or dental courses. Students from lower SEBs might thus view dental applications as excessively risky and hence prefer to utilise all their choices to apply to less competitive courses.^26^

The meritocratic discourse explored in the current study strongly dominated and overpowered alternative counter-discourses, underlining the importance of WA for diversity expansion within the dental workforce and improved patient-care delivery. The degree of this dominance was unexpected due to raising recognition of this phenomenon internationally.^22^^,^^33^ Consistently, alternative characteristics recognised within WA applicants were only occasionally mentioned and as such, excluded and not communicated explicitly as valuable. Students from lower SEBs can: better understand cultures of patients from similar backgrounds; appreciate needs of underserved patient-populations; and are more likely to undertake unpopular career niches or fill less attractive posts.^34^ Only two HEIs commented very briefly on the benefits applicants from lower SEBs might bring to wider society and no webpage or policy document echoed the positive impact of those applicants on the dental profession and patient care.

Different discourses can affect applicants in different ways. For instance, on one dental school website, WA initiatives were offered to prospective students as a form of support to compensate for their challenging circumstances which could prevent them from reaching their maximal potential during admission process. Thus, applicants can perceive this as a chance to study their chosen degree, thereby experiencing a sense of social justice. However, those individuals might feel more vulnerable due to needing external voluntary support. However, another dental school positioned their WA initiative as a way of training a workforce benefiting the wider society. The latter discourse might appear more empowering to WA applicants.

Our results are also in agreement with Razack *et al.*^35^ where HEIs displayed a preference for candidates exhibiting high academic attainment. However, the discourses on diversity in Canada were presented as a pinnacle of 'multicultural sophistication' of HEIs, with equity being viewed as enhancing participation of learners from rural areas.

To enact WA policy, various HEIs introduced a variety of strategies, including: outreach activities; mentoring schemes; bursaries; summer schools; or alternative WA entry routes with lower entry qualifications.^36^ Certain school websites also contained personal success stories of students who took part in WA initiatives and expressed their gratitude and indebtedness for receiving an opportunity to study dentistry. Earlier research has demonstrated the positive influence of role models on encouraging applications from WA students to study clinical degrees.^37^^,^^38^

Except for financial implications, administration of these additional measures can have other consequences for the universities involved. Publicly available rankings of HEIs are compiled deriving data from multiple sources, including entry standards and completion rates.^39^ High position within a ranking not only allows a HEI to access higher external funding, but also increases its popularity among highly qualified learners who are particularly attracted to prestigious HEIs; thus, creating a self-reinforcing mechanism.^40^ Dental schools might be disheartened to recruit WA students with lower attainment levels due to a perceived negative impact on their ranking positions and increased risk of student attrition. If societal benefits do not align with institutional characteristics promoted by media ranking, HEIs could deviate from conduct, which is socially desirable.^41^

To effectively support their WA applicants, dental schools might want to consider the suggestions presented in Table 1 and incorporate them into their local protocols. This will allow them to select high-quality candidates using research-supported methods, rather than relying on heuristics.^42^

**Table 1:** Strategies enhancing enactment of local WA policy

Pre-application phase	Application phase	Post-application phase
Outreach programmes for school leavers^43^	Contextual admissions^6^	Allocation of dedicated places^19^
Access-to-dentistry courses for WA applicants^44^	Evidence-based selection methods^42^	Mentoring to decrease attrition risk^48^
Discourses on websites underlying benefits for the profession and patients^45^		
Role models^46^		
Mentoring schemes^47^		

### Limitations

The present study employed one data type (written text) in order to produce a focused analysis. Exclusion of images and layout can narrow available perspective and restrict contextualisation within a larger pool of information. As certain websites contained video- and audio-recordings, triangulation of different information sources might have augmented the credibility of the drawn interpretations. 

Similarly, this study focused on examination of one information material genre: websites of schools with associated documents. Future research might include additional genres, such as: prospectuses; leaflets; open-days field notes; or semi-structured interviews with admission officers.^24^^,^^27^^,^^28^^,^^35^

## Conclusion

In summary, we found a commitment to WA on the websites of most UK dental schools. However, the presentation and value of WA was restricted to a narrow range of definitions. To truly realise the benefits of WA, dental schools should consider portraying an additional discourse: emphasising the benefits that a wider range of learners can bring into dentistry and patient care. This may convince them about their suitability for a career in dentistry, with resultant benefits to patients and the entire profession.
